# The antibacterial and angiogenic effect of magnesium oxide in a hydroxyapatite bone substitute

**DOI:** 10.1038/s41598-020-76063-9

**Published:** 2020-11-05

**Authors:** Catarina C. Coelho, Tatiana Padrão, Laura Costa, Marta T . Pinto, Paulo C. Costa, Valentina F. Domingues, Paulo A. Quadros, Fernando J. Monteiro, Susana R. Sousa

**Affiliations:** 1 i3S - Instituto de Investigação e Inovação em Saúde, Universidade do Porto, Rua Alfredo Allen, 208, 4200-135 Porto, Portugal; 2 INEB - Instituto de Engenharia Biomédica, Universidade do Porto, Rua Alfredo Allen, 208, 4200-135 Porto, Portugal; 3 FEUP - Faculdade de Engenharia, Universidade do Porto, Rua Dr. Roberto Frias, s/n, 4200-465 Porto, Portugal; 4 FLUIDINOVA, S.A., Rua Engenheiro Frederico Ulrich, 2650, 4470-605 Maia, Portugal; 5 ISEP - Instituto Superior de Engenharia do Porto, Instituto Politécnico do Porto, Rua Dr. António Bernardino de Almeida, 431, 4200-072 Porto, Portugal; 6 IPATIMUP – Instituto de Patologia e Imunologia Molecular, Universidade do Porto, Rua Júlio Amaral de Carvalho, 45, 4200-135 Porto, Portugal; 7 UCIBIO/REQUIMTE, MEDTECH, Laboratório de Tecnologia Farmacêutica, Departamento de Ciências do Medicamento, Faculdade de Farmácia, Universidade do Porto, Rua de Jorge Viterbo Ferreira, 228, 4050-313 Porto, Portugal; 8 REQUIMTE/LAQV/GRAQ, Instituto Superior de Engenharia do Porto, Instituto Politécnico do Porto, Rua Dr. António Bernardino de Almeida, 431, 4200-072 Porto, Portugal

**Keywords:** Biomedical materials, Biomaterials

## Abstract

Bone graft infections are serious complications in orthopaedics and the growing resistance to antibiotics is increasing the need for antibacterial strategies. The use of magnesium oxide (MgO) is an interesting alternative since it possesses broad-spectrum antibacterial activity. Additionally, magnesium ions also play a role in bone regeneration, which makes MgO more appealing than other metal oxides. Therefore, a bone substitute composed of hydroxyapatite and MgO (HAp/MgO) spherical granules was developed using different sintering heat-treatment cycles to optimize its features. Depending on the sintering temperature, HAp/MgO spherical granules exhibited distinct surface topographies, mechanical strength and degradation profiles, that influenced the in vitro antibacterial activity and cytocompatibility. A proper balance between antibacterial activity and cytocompatibility was achieved with HAp/MgO spherical granules sintered at 1100 ºC. The presence of MgO in these granules was able to significantly reduce bacterial proliferation and simultaneously provide a suitable environment for osteoblasts growth. The angiogenic and inflammation potentials were also assessed using the in vivo chicken embryo chorioallantoic membrane (CAM) model and the spherical granules containing MgO stimulated angiogenesis without increasing inflammation. The outcomes of this study evidence a dual effect of MgO for bone regenerative applications making this material a promising antibacterial bone substitute.

## Introduction

Bone is a complex, vascularized and highly dynamic tissue, capable of remodelling and self-healing throughout life^1^. However, bone disorders such as trauma, tumours, infections, congenital disorders and other conditions may affect its homeostasis^2^. Addressing the bone defects created by these situations is a serious challenge for clinicians that often rely on bone grafting strategies. In fact, bone is the second most transplanted tissue worldwide, with 2.2 million procedures performed each year with an expected annual growth of 13%^3^. The massive demand for bone grafting materials is not only related with advances in healthcare services and consequently increased life expectancy^4^, but also with increased obesity and insufficient physical activity of an elderly population^5^. Synthetic bone substitutes are probably the best solution for bone grafting since they are completely safe and readily available, in opposition to autografts, allografts and xenografts^2,6^. Hydroxyapatite (HAp)-based bone substitutes have been successfully used over the last decades. Their success is probably related with HAp chemical similarity with natural bone, resulting in excellent biocompatibility, bioactivity and osteointegration^7,8^. However, the main limitation of calcium-phosphates such as HAp is their brittleness and poor mechanical strength^9^.

The growth of bone grafting procedures will necessarily correlate with an increase of bone graft infections due to contamination on the surgical site. Implant-associated infections are one of the most frequent and severe problems related with the use of biomaterials^10,11^. In the USA, these infections represent 25.6% of all healthcare associated infections^12^. The majority of implant-associated infections in orthopaedics is caused by *Staphylococcus aureus* (*S. aureus*) and *Staphylococcus epidermidis* (*S. epidermidis*), together representing two thirds of those infections. After staphylococci, *Pseudomonas aeruginosa* (*P. aeruginosa*) is one of the most relevant in terms of prevalence^13,14^. These infections are highly resistant to antibiotic therapy, often requiring the removal of the device and implantation of a revision one^15^. Considering the relevance of implant-associated infections, it is mandatory to develop bone grafts capable of inhibiting bacterial colonization and numerous strategies have emerged in recent years. In particular, antibiotic-loading materials became a common approach for prevention and treatment of implant-associated infections^16^. However, this approach is becoming limited by the occurrence of the antibiotic resistance crisis^13,17^. The use of metal oxides may be a good alternative since they have large antibacterial spectra and high stability^18,19^. Specifically, magnesium oxide (MgO) is a very interesting option to develop antibacterial bone grafts as it possesses strong antibacterial activity and it is cost-effective^20,21^. In fact, a previous work from our group showed that the inclusion of MgO reduced bacterial growth and biofilm formation in a HAp-based bone substitute^22^. Moreover, materials containing magnesium ions were proved to be beneficial for bone regenerative strategies since this element improved the healing of bone defects. Namely, it has been shown that magnesium improves osteoblasts differentiation and proliferation^23,24^. Magnesium also plays a role in stimulating endothelial cells recruitment and inflammation. It is also important to mention that MgO is one of the magnesium compounds recognized as safe by the Food and Drug Administration (FDA)^25^.

The main challenge to develop an antibacterial bone graft is to achieve the best antibacterial performance along with suitable tissue interactions and biocompatibility^26^. Additionally, these materials must have appropriate mechanical strength not only for being manipulated during implantation but also for the process of packaging and transportation, since this type of products usually may undergo partial erosion and can even break apart^27^. Moreover, an easy surgical implantation is also very important for the surgeon and the patient. The use of spherical particles allow a more controlled flowability during implantation and the possibility of using minimal invasive procedures, when compared to irregular particles^28^.

In this study, MgO was added to HAp spherical granules to combine its antibacterial activity together with the potential benefit of magnesium ions to the regeneration process. HAp/MgO spherical granules were produced using different sintering heat-treatment cycles to achieve adequate mechanical stability. For that purpose, mechanical characterization was performed, namely compressive strength and friability. Furthermore, degradation behaviour was assessed according to ISO 10,993–14:2001 to study the chemical degradation and also in the presence of simulated body fluid (SBF) to evaluate biodegradation and apatite formation. As different sintering heat-treatments can originate distinct structures and surfaces, the influence of the sintering temperature in their biological performance was evaluated, particularly in terms of antibacterial activity and on initial bacterial adhesion to the surface. Cytocompatibility was also evaluated by assessing the metabolic activity of osteoblasts and their ability to adhere onto the materials. Finally, angiogenic potential and inflammation were assessed using the in vivo chick embryo chorioallantoic membrane (CAM) model.

## Results

### Spherical granules characterization

**Figure 1:**
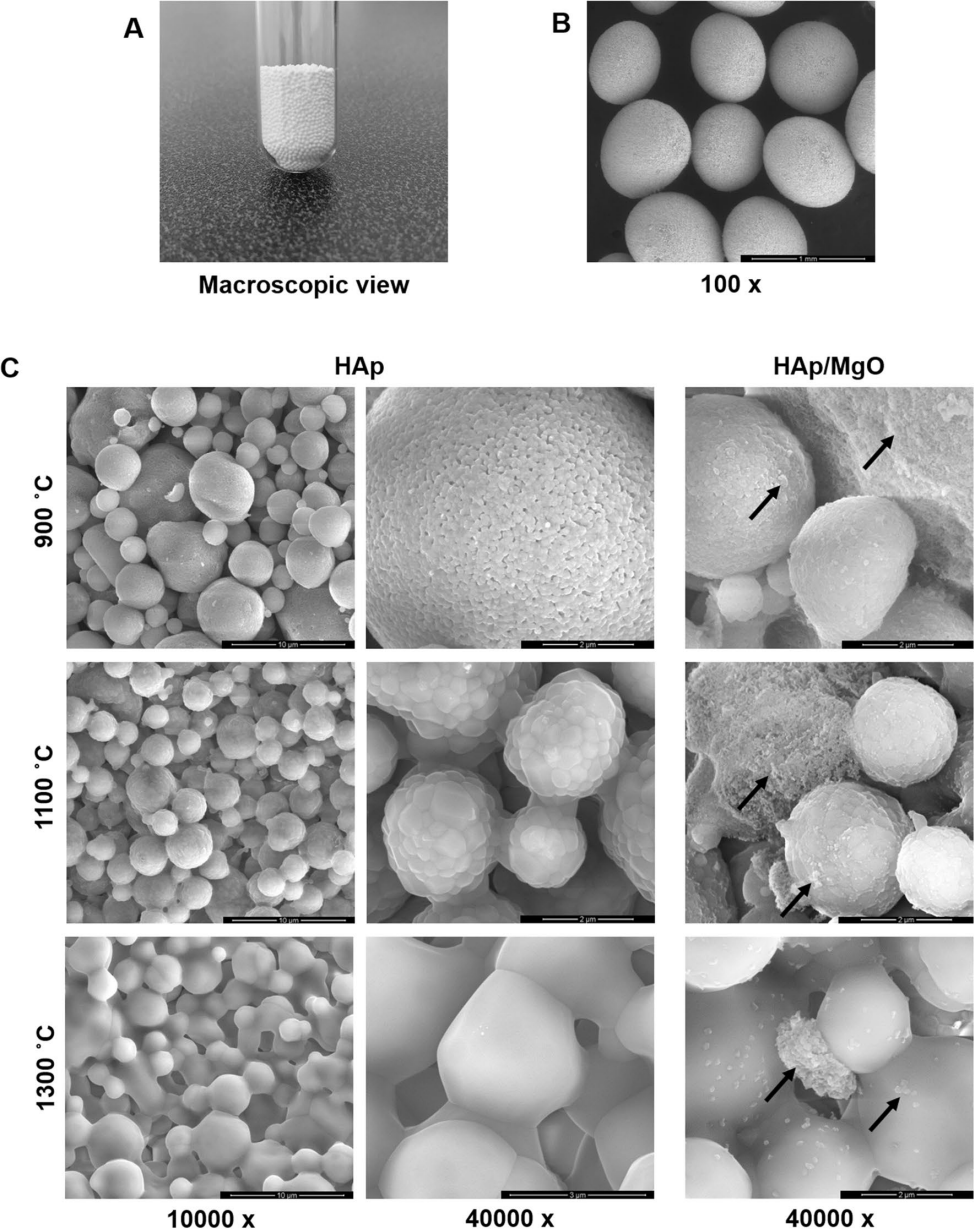
Morphology and surface of the spherical granules. (**A**) Macroscopic view of the spherical granules; (**B**) General view of the granules acquired with SEM evidencing their spherical morphology and size; and (**C**) SEM images showing the surface details of HAp and HAp/MgO spherical granules sintered at 900, 1100 and 1300 °C. Arrows point out the MgO particles enclosed in the middle of the HAp ones.

SEM was used to observe the effect of the different heat-treatments on spherical granules surface (Fig. 1). Figure 1A,B show general views of the granules, evidencing their size and spherical shape. Both HAp and HAp/MgO granules had the same macroscopic appearance and size range. The surface details of both HAp and HAp/MgO spherical granules are presented in Fig. 1C, in which it can be also observed that higher sintering temperatures lead to the coalescence of HAp particles. This is particularly evident for the materials sintered at 1300 °C. For higher sintering temperatures, granules also possess bigger HAp crystals, compared to lower temperatures. Spherical granules sintered at 900 °C also presented nanoporosity. This characteristic of the surface resulted from the powder used as raw material and it vanished when the material was sintered at 1300 °C. Regarding the HAp/MgO spherical granules, the MgO was enclosed in the middle of the HAp as bigger aggregates composed of MgO nanoparticles. Additionally, it was also found as smaller particles, spread over the surface and on top of the HAp. The MgO nanopowder used for the production of HAp/MgO granules maintained its nanostructure even when higher sintering temperatures were used.

**Figure 2:**
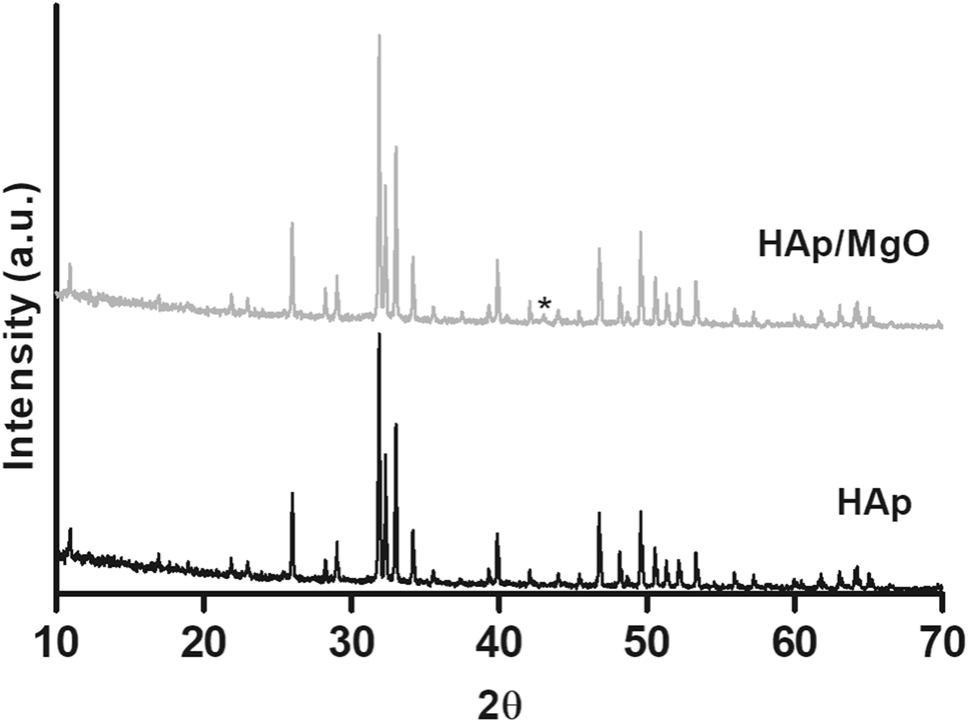
XRD spectra obtained for HAp and HAp/MgO spherical granules sintered at 1100 °C. *Represents a peak at 43° that corresponds to MgO.

In Fig. 2, XRD spectra for HAp and HAp/MgO spherical granules sintered at 1100 °C may be observed. The inclusion of MgO and the increase of the sintering temperature did not cause the appearance of other calcium phosphate phases. Same results were obtained for spherical granules sintered at 900 °C and 1300 °C (data not shown).

**Figure 3:**
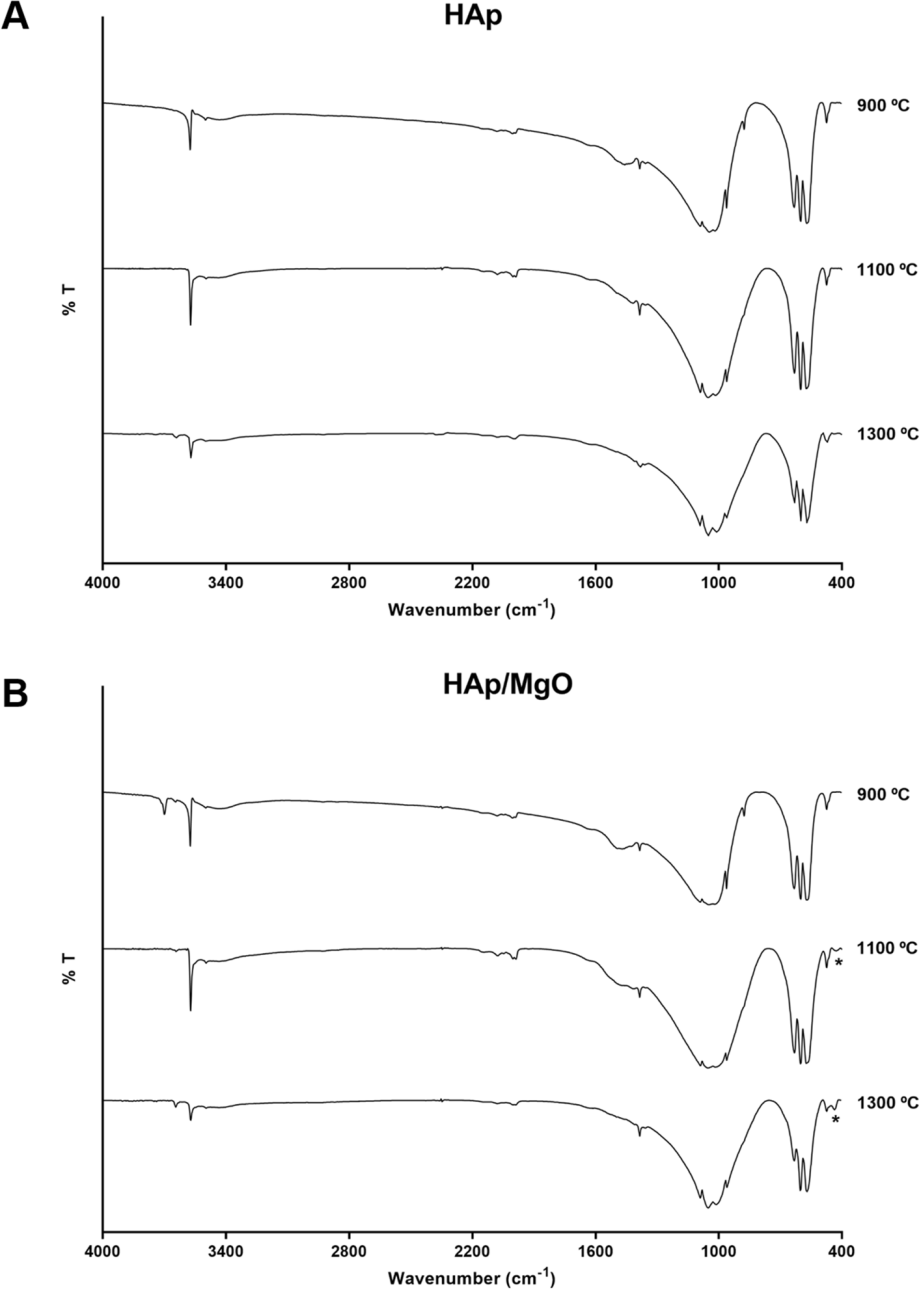
FTIR spectra of HAp and HAp/MgO spherical granules sintered at 900, 1100 and 1300 °C. * Indicates the vibration of Mg-O.

Figure 3 illustrates the FTIR spectra of HAp and HAp/MgO spherical granules sintered at different temperatures. For all materials, the presence of HAp characteristic peaks is clear, namely phosphate groups (474, 570, 601, 962, 1045 and 1090 cm^−1^) and hydroxyl groups (633 and 3573 cm^−1^). Mg-O peak (436 cm^−1^) is not evident for 900 °C but it becomes clear for 1100 °C and 1300 °C since unresolved bands become sharper due to high crystallinity. Splitting peaks in the phosphate bound around 1000 cm^−1^ also indicates higher crystallinity. Carbonate groups are also present in HAp and HAp/MgO spherical granules sintered at 900 °C (regions 800–900 and 1400–1480 cm^−1^) but these peaks tend to disappear at 1100 and 1300 °C. In the HAp/MgO samples, a peak related with the stretching mode of OH^-^ groups can be found around 3700 cm^-1^ that can be related with the presence of Mg(OH)_2_^29^_._

**Figure 4:**
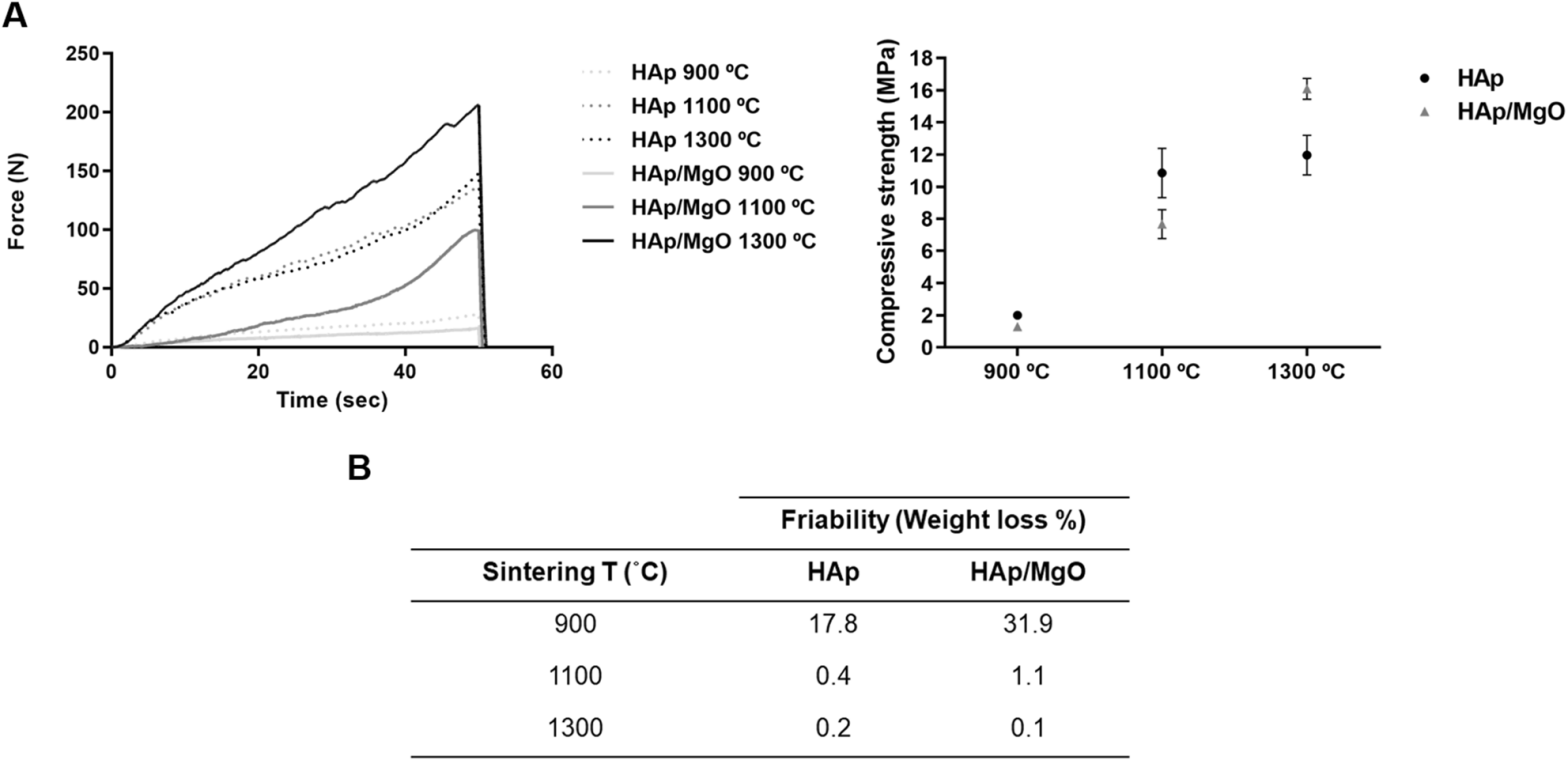
Mechanical properties of the HAp and HAp/MgO spherical granules. (**A**) Force–time curves obtained for each material and compressive strength calculated from the parameters obtained by force–time curves; (**B**) Friability results expressed in terms of weight loss.

Compression and friability tests were performed to evaluate the mechanical properties of the spherical granules (Fig. 4). As expected, the increase of the sintering temperature resulted in materials with superior mechanical strength (Fig. 4A). For the same sintering temperature, the spherical granules containing MgO had inferior mechanical strength in comparison with pure HAp, except for materials sintered at 1300 °C (Fig. 4A). The friability results are in accordance with the ones obtained in the compression test. Higher weight loss was obtained for spherical granules sintered at 900 °C (Fig. 4B). Particularly, the HAp/MgO spherical granules sintered at 900 °C presented a friability value of 31.9%. Granules containing MgO also presented lower friability values, when compared with pure HAp materials, except for the materials sintered at 1300 °C.

### Chemical degradation and biodegradation/apatite formation

**Figure 5:**
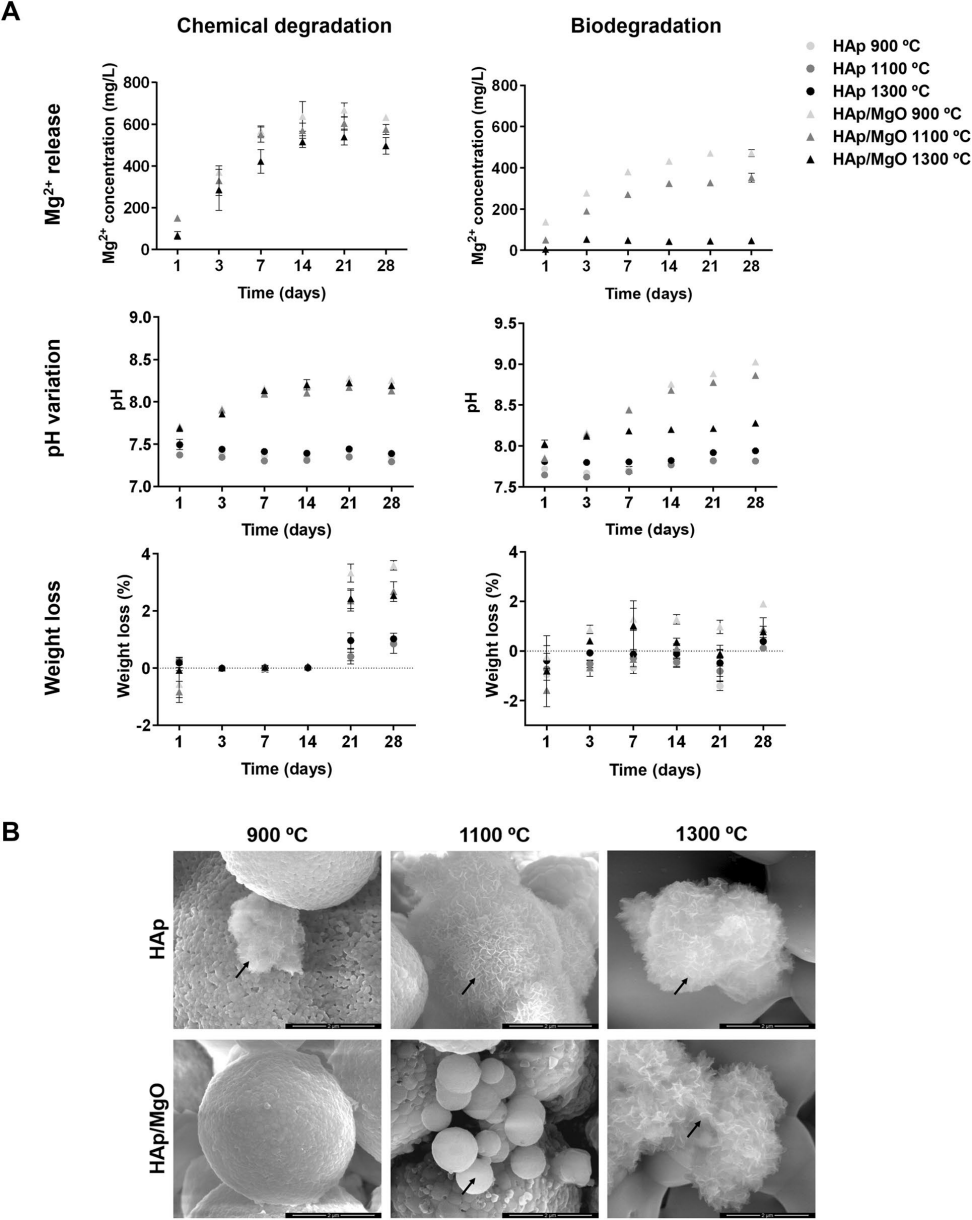
Chemical degradation and biodegradation assays performed with HAp and HAp/MgO spherical granules for 28 days. A) Mg^2+^ release, pH variation and weight loss over time for the different materials; B) SEM images illustrating apatite formation on granules surface after 28 days of immersion in SBF (black arrows) (Scale bar = 2 µm; 50,000 × magnification).

The chemical degradation of the different materials was evaluated according to ISO 10993-14:2001 standard using Tris–HCl as immersive media. Additionally, the apatite formation on top of the materials was also investigated by immersing the spherical granules in SBF. For both tests, degradation behavior was studied in terms of Mg^2+^ release, pH and weight loss (Fig. 5A) and the apatite formation was visualized by SEM (Fig. 5B). Figure 5A depicts the Mg^2+^ concentration over immersion time and it was observed that an increase in sintering temperature resulted in lower amounts of this ion in solution, for both assays. However, this difference is much more noticeable in the case of SBF immersion, especially for the HAp/MgO granules sintered at 1300 °C. In terms of pH change, the inclusion of MgO increased pH for both degradation assays. Similar pH increase was observed for the HAp/MgO spherical granules in the chemical degradation assay, as opposed to the SFB assay in which pH changes were more significant. In the chemical degradation assay, weight loss was only observed after 21 days and HAp/MgO spherical granules showed higher weight loss than pure HAp granules, especially for the materials sintered at 900 °C. Pure HAp granules showed very similar weight loss for all sintering temperatures. For the biodegradation assay, HAp/MgO granules sintered at 900 °C also showed the highest weight loss. Regarding the apatite formation with SBF, it was possible to observe apatite formation after 28 days in all spherical granules tested except for HAp/MgO sintered at 900 °C (Fig. 5B). For pure HAp granules sintered at 900 °C, the clusters of new apatite formed on top of the surface were also smaller. It was also evident that the apatite formed on HAp/MgO spherical granules sintered at 1100 °C had a completely different appearance from the morphology observed in the rest of the materials.

### Antibacterial activity

**Figure 6:**
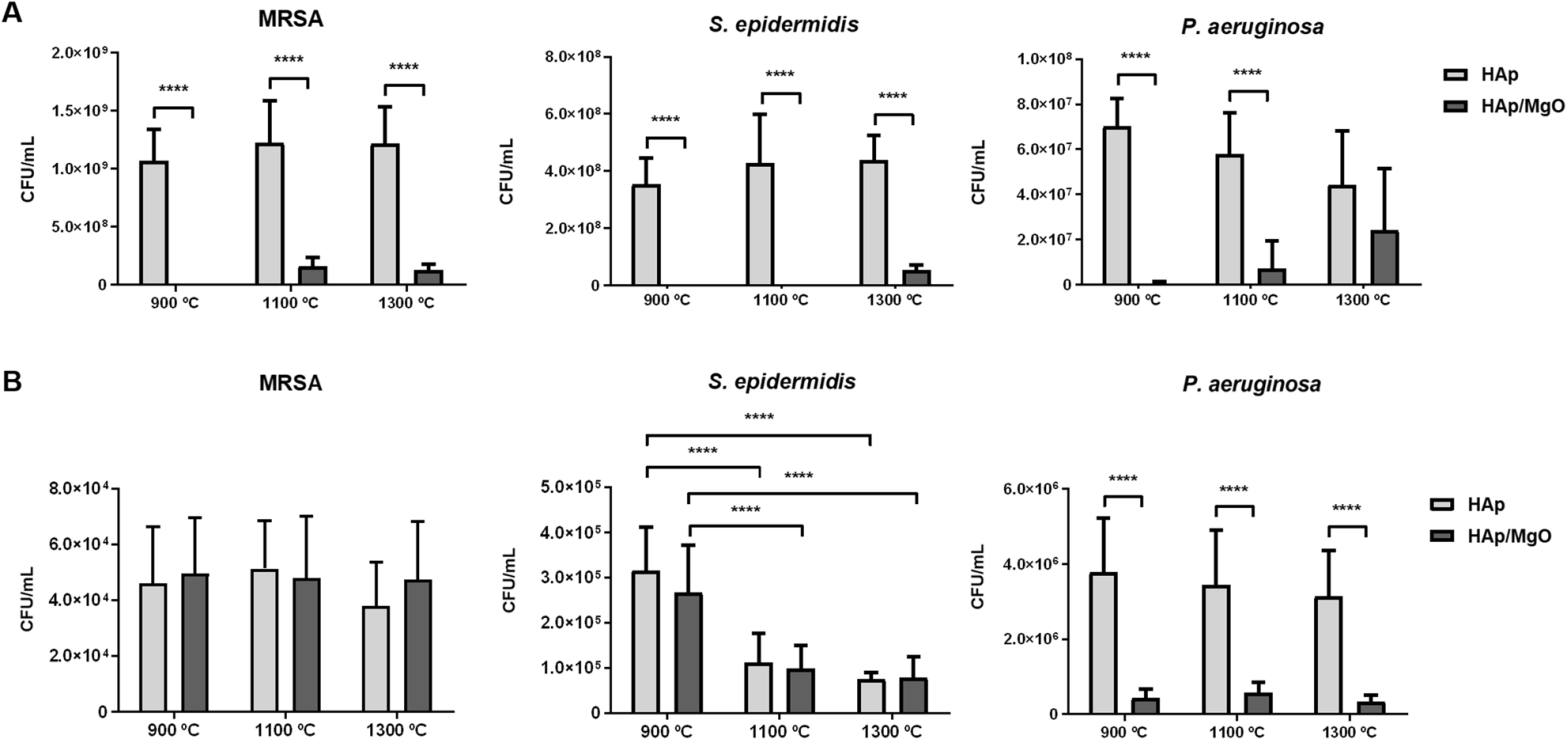
Antibacterial activity of the HAp and HAp/MgO spherical granules. A) Planktonic growth after 24 h incubation with the spherical granules; B) Initial bacterial adhesion onto the surface after 1 h incubation with the spherical granules. ****Indicates a statistical significant difference (p < 0.0001).

The antibacterial activity was evaluated in terms of planktonic bacteria and initial bacterial adhesion. For all bacterial strains tested, HAp/MgO spherical granules were able to significantly reduce planktonic growth after 24 h incubation, except for materials sintered at 1300 °C towards *P. aeruginosa* (Fig. 6A). In terms of initial bacterial adhesion, it can be observed that the increase in sintering temperature led to lower adhesion of *S. epidermidis* onto the spherical granules, with no differences between pure HAp and HAp/MgO granules (Fig. 6B). On the contrary, there was no effect of the sintering temperature in initial adhesion for *P. aeruginosa* as it can be observed in Fig. 6B*.* For this strain, the presence of MgO reduced the initial adhesion for all temperatures, compared to pure HAp granules. For methicillin-resistant *S. aureus* (MRSA), neither the sintering temperature nor the presence of MgO in the samples affected the initial bacterial adhesion onto the spherical granules (Fig. 6B).

### In vitro cytocompatibility

**Figure 7:**
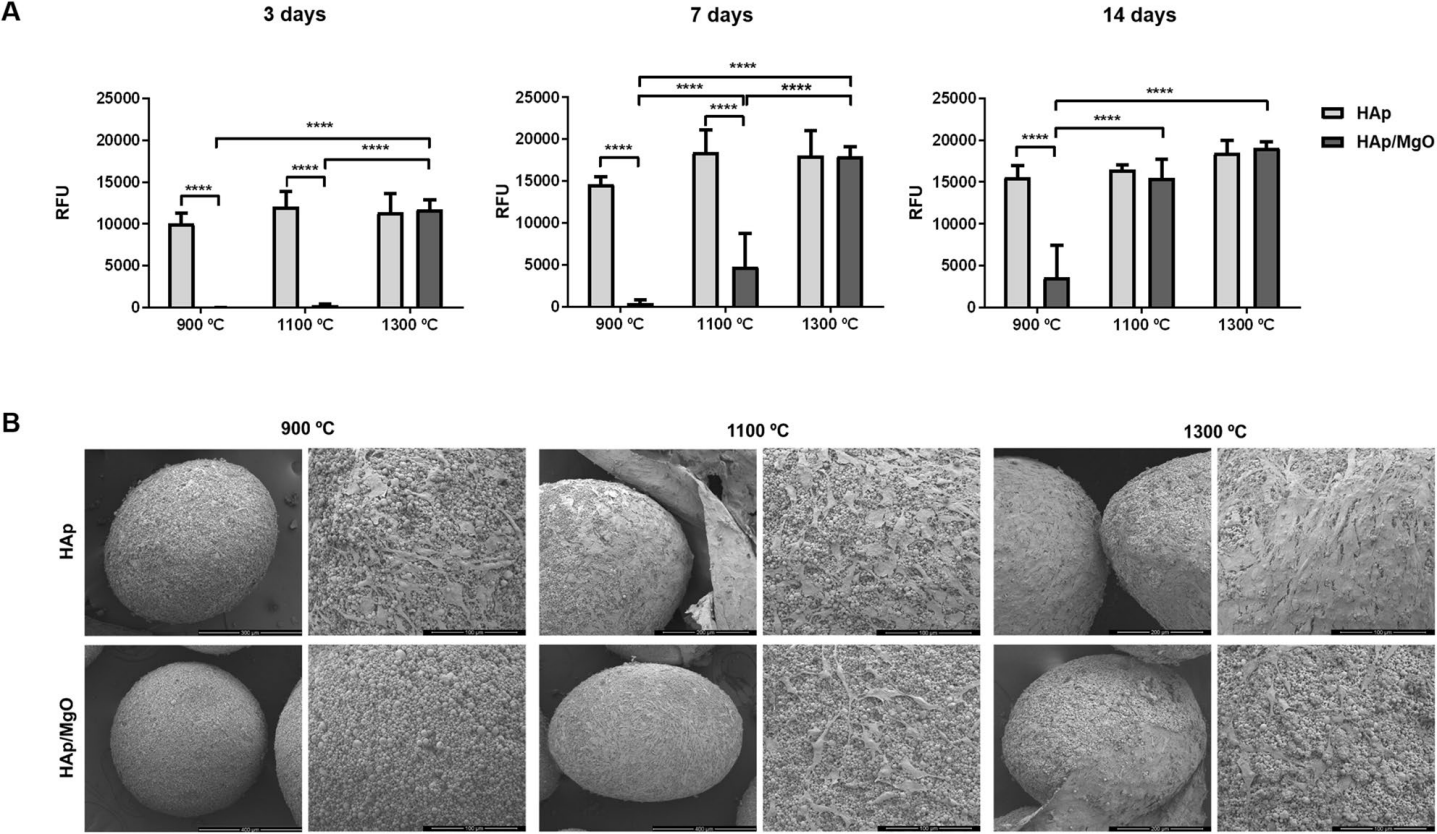
Cytocompatibility assay with MC3T3-E1 cells cultured with HAp and HAp/MgO spherical granules. (**A**) Metabolic activity of the MC3T3-E1 cells after 3, 7 and 14 days of culture. TCPS were used as a control (data not shown); (**B**) SEM images of the cells adhered on the materials, with a general view of the materials on the left side and a detailed of the surface on the right side. ****Indicates a statistical significant difference (p < 0.0001).

Concerning the in vitro cytocompatibility tests, MC3T3-E1 cells seeded on HAp/MgO spherical granules sintered at 900 and 1100 °C showed a significantly lower metabolic activity at day 3 and day 7, when compared to pure HAp granules for the same temperature and to HAp/MgO granules sintered at 1300 °C (Fig. 7A). However, after 14 days of culture, only the HAp/MgO spherical granules sintered at 900 °C continued to have a significantly lower metabolic activity. SEM images were obtained to observe cell behavior on granules (Fig. 7B). After 14 days of culture, HAp/MgO spherical granules sintered at 900 °C did not present adhered cells on top the materials. In opposition, all other granules presented higher number of cells adhered on the surface, with some regions containing individual cells but others regions were totally covered with cells.

### In vivo CAM assay

**Figure 8:**
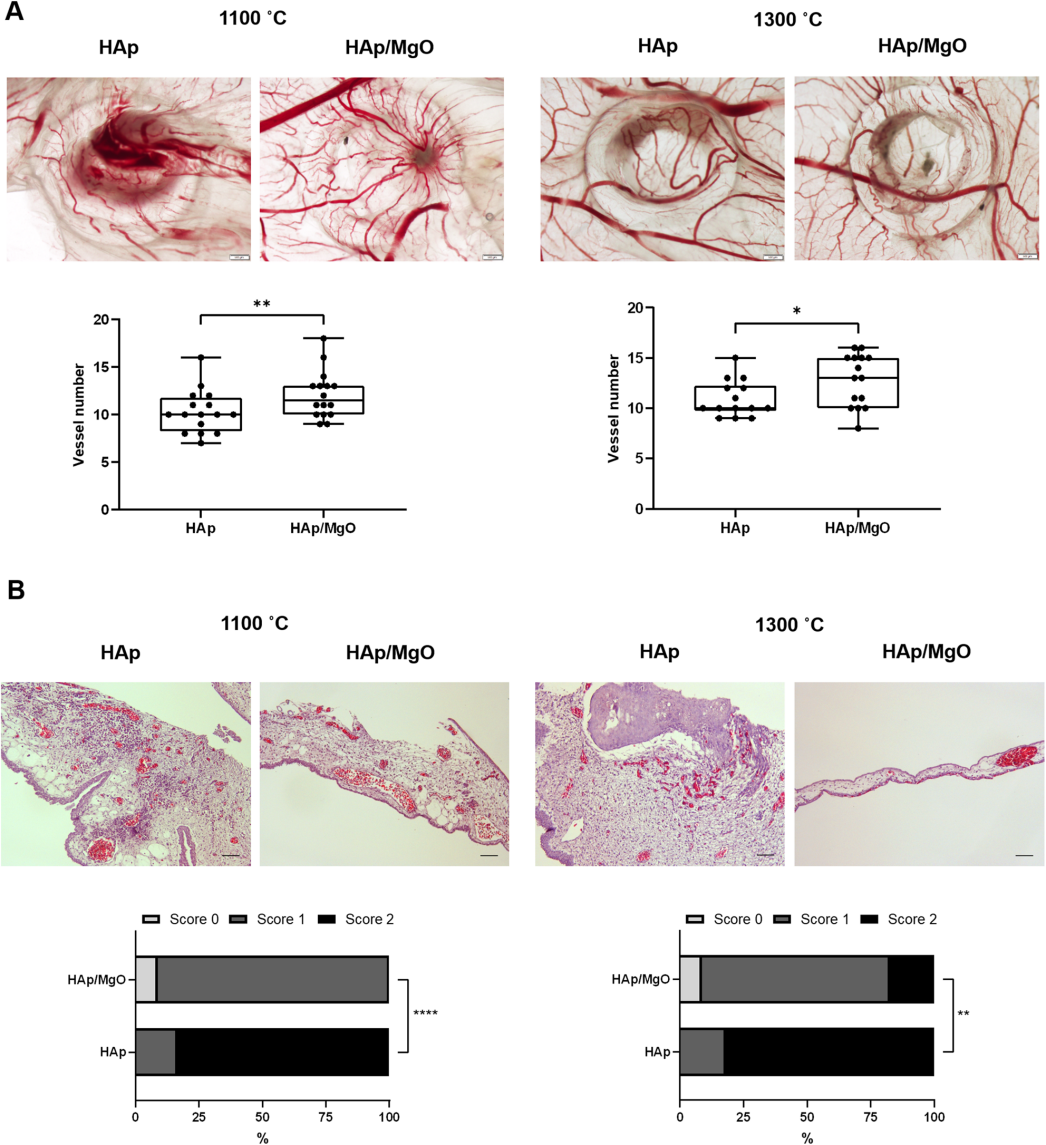
In vivo CAM assay performed with HAp and HAp/MgO spherical granules sintered at 1100 and 1300 °C. (**A**) Representative images used to determine the angiogenic response of the materials and the quantification of neovessels; (**B**) Inflammation score analysis made on macroscopic images of excised CAMs [Score 0 = no inflammatory reaction. Score 1 = with inflammatory reaction. Score 2 = with inflammatory reaction, bigger than the counterpart (in the same egg)]. Histological images of the CAM stained with H&E validated the inflammation scores, with an inflammatory response characterized by dilation of the CAM (Scale bar = 100 µm; 10 × magnification). * represents a statistically significant difference with p < 0.05; **p < 0.01 and ****p < 0.0001.

The in vivo CAM model was used to evaluate the angiogenic potential and the inflammatory response of the spherical granules. Since the materials sintered at 1100 and 1300 °C presented the most appropriate mechanical properties and cytocompatibility, only these were considered for this assay (Fig. 8). The results showed that HAp/MgO granules had significantly higher angiogenic response for both sintering temperatures, when comparing to pure HAp granules (Fig. 8A). Moreover, the granules containing MgO also revealed lower inflammatory response (Fig. 8B). Comparing granules sintered at 1100 and 1300 °C, no differences were observed in terms of angiogenic potential and inflammation for each type of granules (data not shown).

## Discussion

In this study, the properties of an antibacterial HAp/MgO spherical bone substitute were tuned to optimize its performance. With that purpose, three different sintering temperatures were tested and their effect on the spherical granules properties was evaluated. The main purpose of changing the sintering temperature was to obtain a bone substitute with sufficient mechanical strength to overcome handling and transportation, since this is a key factor for its success. It was also critical to evaluate the cytocompatibility of the materials given that MgO may be toxic to mammalian cells agent^30^. For all those reasons, it is crucial to achieve a balance between mechanical stability, antibacterial activity and cytocompatibility.

The use of a granular bone substitutes allows its application in bone defects with distinct sizes and shapes, overcoming the limitation of bone grafts with predefined shapes^31^. A spherical morphology also eases surgical implantation allowing the use of minimally invasive techniques with a more controlled flowability. Additionally, sphericity improves packing within the defect and the generation of a regular interstitial intergranular macroporosity that promotes efficient osteoconduction^28,32^. On the contrary, irregular particles create a more heterogeneous packing inside the defect that may impair new bone ingrowth and vascularization, as well as increase inflammatory response^33,34^.

As observed with SEM, the increase in sintering temperature resulted in HAp crystals coalescence and consequently lower microporosity. This also results in increased and stronger contact between the aggregates of crystals resulting from partial fusion between them. As expected, increasing the sintering temperature resulted in significantly higher compressive strength. For sintering at 900 °C and 1100 °C, pure HAp spherical granules had higher mechanical strength than HAp/MgO materials. This is probably related with differences in HAp and MgO particles, as they have different sizes and morphologies that may result in defective particle packing and a less cohesive structure. On the contrary, for sintering 1300 °C HAp/MgO spherical granules presented superior mechanical strength, when compared with HAp ones. Besides the increased contact between HAp and MgO, it has also been reported that the presence MgO can improve the mechanical resistance of HAp-based materials^35^. At this temperature, MgO is not sintered but the HAp sintering produces the blocking of MgO particles within the structure of HAp.

In the pharmaceutical industry, friability test is an accepted standard for determining the resistance of tablets to scratch and shock that happens during the production, packing and shipping processes. Normally, bone substitutes are used for non-load bearing applications but they must be able to resist to these same processes, as well as manipulation during surgery. Therefore, friability analysis was performed to evaluate the capacity of the granules to withstand those processes in accordance with standardized figures. The friability results were in agreement with the compressive strength ones, since increasing the sintering temperature resulted in inferior weight loss. Although friability limits are not established for these tested materials, it is recommended that weight difference do not exceed 1%^27^. Spherical granules sintered at 900 °C had friability values that surpass by far that limit, while the materials sintered at 1100 and 1300 °C presented weight losses very close or below 1%.

The results obtained in the degradation assays were in agreement with the mechanical characterization. As expected, increasing the sintering temperature resulted in lower amounts of Mg^2+^ being released since the MgO particles became more entrapped in the HAp structure. The amount of Mg^2+^ released was also dependent on the immersive media. When in SBF, the differences on Mg^2+^ release were quite evident between the materials, with lower concentrations of Mg^2+^ comparing to degradation in Tris–HCl. Nevertheless, it is important to refer that the Mg^2+^ concentration released from the HAp/MgO spherical granules sintered at 900 and 1100 °C were within the concentration range reported to induce osteogenic differentiation^36,37^. Spherical granules containing MgO also caused a pH increase over time, which may be easily explained due to the hydroscopic nature of MgO that forms Mg(OH)_2_ when exposed to water^38^. Then, Mg(OH)_2_ dissociated and released Mg^2+^ and OH^-^^39^. The immersive media also influenced the increase of pH caused by HAp/MgO spherical granules, with a higher pH values observed when immersed in SBF. Finally, higher values of weight loss were observed for the HAp/MgO granules sintered at 900 °C, both in Tris–HCl and SBF. The weight loss in SBF was not consistent over time as expected since this medium possesses different ions in its composition and allows the new precipitation of apatite. In terms of biodegradation, it was possible to observe that all materials allowed the formation of new apatite, except for the HAp/MgO spherical granules sintered at 900 °C. The immersion of HAp/MgO spherical granules into SFB caused a pH increase which decreases HAp solubility and consequently accelerates apatite nucleation^40^. Therefore, it could be expected apatite formation for the HAp/MgO spherical granules sintered at 900 °C, which did not occur. It is also known that magnesium influences mineralization both in vitro and in vivo, especially inhibiting crystal growth^41,42^. In fact, Barrere et al. studied the role of magnesium in the formation of Ca-P coatings with SFB and observed that when Mg^2+^ content is increased, Ca-P precipitation is delayed and the precipitate is changed to amorphous or poorly crystallized apatite^43^. Another study also reports that magnesium slows down apatite crystallization but it also points out that the intensity of this effect depends on the concentration of magnesium present^44^. This may explain the results obtained in this work since the amount of magnesium released to the media decreases with the sintering temperature. The HAp/MgO granules sintered at 900 °C showed superior degradation and therefore higher concentration of Mg^2+^ released to the medium, with no apatite formed after 28 days immersion on SBF. In opposition, the HAp/MgO granules sintered at 1100ºC presented dense and smooth particles that probably are apatite precursor spheres^45^. With lower Mg^2+^ release, the HAp/MgO granules sintered 1300 °C showed new apatite formation on their surface with the typical plate-like morphology. It is relevant to refer that the chemical interactions of MgO with physiological fluids are very complex since these present calcium, phosphate, carbonate, other ions and proteins^24^.

The antibacterial activity of the HAp/MgO spherical granules was evaluated towards three of the most relevant bacteria detected in orthopaedic infections, in terms of planktonic growth and initial bacterial adhesion. It was observed that HAp/MgO materials sintered at 900 and 1100 °C were capable to significantly reduce bacterial growth for all the strains tested, in comparison with pure HAp spherical granules. These results are in agreement with other studies that report the effectiveness of MgO in inhibiting both Gram-positive and Gram-negative bacteria^22,46–48^. Aničić et al*.* studied the effect of calcination temperature on the morphological and antibacterial characteristics of MgO microrods^49^. The authors observed that calcination temperatures higher that 700 °C caused the nano-texturing of MgO, which resulted in superior antibacterial activity since it enhanced the contact with bacteria. This finding reinforces the existence of contact-based mechanism for the antibacterial activity of MgO. However, in our study, a tendency to have a decrease in the antibacterial activity for planktonic bacteria was observed with increasing sintering temperature. Although some differences in the nano-texturing of the MgO may happen at higher temperatures, the decrease of antibacterial activity is essentially related to higher stability of the materials, with superior mechanical integrity and slower degradation profiles. Thus, MgO particles are less likely to be released and interact with bacteria. The mechanism of antibacterial action of MgO and other metal oxides is still debated. There are several hypotheses described in the literature but the most accepted mechanism is the formation of reactive oxygen species (ROS). These species chemically attack the bacterial cell membrane causing its disruption and consequent cell death^18,20,50^. The high pH values and the presence of Mg^2+^ had also been proposed to contribute to MgO antibacterial activity^51^. However, it was also shown that *S. epidermidis* and *E. coli* can proliferate significantly up to pH 10^24^. Another study showed that Mg^2+^ alone had no effect in bacterial growth. The authors compared MgO with MgCl_2_ using the same concentration of magnesium and MgCl_2_ did not affect bacterial growth, suggesting an antibacterial mechanism involving an oxygen radical generated from the MgO^52^. It is possible that the antibacterial mechanism of MgO is a combination of all these factors and, depending on the bacterial strain, some of them may prevail.

Initial bacterial adhesion to surfaces is a critical phase in the pathogenesis of medical devices infection that can lead to biofilm formation. This phase occurs in the first hours and depends on the nature of the biomaterial and on the cell surface characteristics of the bacteria^53,54^. Once established in a biofilm, bacteria become more resistant to host defense and antibiotic treatments^55^. Therefore, it is valuable to better understand initial bacterial adhesion to prevent it. In this work, it was showed that bacterial adhesion to the HAp and HAp/MgO spherical granules is dependent on the bacterial strain. *S. epidermis* showed increased ability to adhere onto materials sintered at 900 °C, with no differences between HAp and HAp/MgO spherical granules. This behavior was already reported in other adhesion studies with HAp and it could be related with morphologic and physical properties of the surface. It is known that increasing the HAp sintering temperature results in smoother surfaces, with less porosity and reduced actual surface area, thus reducing bacterial adhesion^56,57^. Similar behavior was expected for MRSA, since the two strains are Gram-positive coccus bacteria. There is a slight decrease on MRSA adhesion for the granules sintered at 1300 °C, but no significant differences were observed between all materials. As the two strains present similar morphology and size, the difference in terms of initial bacterial adhesion may be related with the chemical nature of their cell walls, as they differ in terms of composition and structure of teichoic acids^58^. Finally, *P. aeruginosa* showed not to be sensitive to morphological changes caused by sintering temperature but rather by the chemical composition of the granules, since the inclusion of MgO significantly reduced its initial adhesion.

In terms of toxicity, it is known that MgO nanoparticles can release significant amounts of magnesium ions but no toxicity was associated to these ions alone^21,59^. In fact, it is strongly believed that the toxicity of MgO nanoparticles is related to ROS production. These chemical species are responsible for inducing DNA damage, lipid peroxidation and protein denaturation. Ghobadian et al. showed that MgO nanoparticles were toxic to zebrafish, inducing cellular apoptosis and intracellular ROS^60^. Other study also showed MgO dose-dependent toxicity towards cancer cells due to ROS generation and further apoptosis^25^. It is important to refer that in these studies MgO was used alone and as individual nanoparticles, which exposure is different from its incorporation on certain biomaterials. The cytocompatibility assay with MC3T3-E1 osteoblasts also showed that MgO caused a deleterious effect on cells metabolic activity for the spherical granules sintered at 900 °C. It was also observed by SEM that cells neither adhered nor proliferated on the top of this material. This result can be explained by the poor mechanical stability and consequently faster degradation profile of this material that increased the amount of MgO released to the media, creating a harsher environment for cell growth. However, by changing the sintering temperature to 1100 and 1300 °C, it was possible to tune the amount of MgO released from the granules and improve their cytocompatibility. Other studies also reported the successful incorporation of MgO into biomaterials for bone regeneration without toxicity. Yuan et al*.* added MgO to poly(lactide-co-glycolide) (PLGA) microspheres and those materials were non-cytotoxic to bone marrow mesenchymal stromal cells^36^. Moreover, it was showed that the incorporation of MgO on a high density polyethylene scaffold did not affect cell proliferation and viability^61^. Another work also evaluated the behaviour of MC3T3-E1 cells on a polycaprolactone/HAp scaffold containing MgO and the authors concluded that HAp and MgO enhanced cell response comparing to pure polymer scaffolds^62^.

Considering the poor mechanical properties and cytocompatibility of the HAp/MgO spherical granules sintered at 900 °C, only the materials sintered at 1100 and 1300 °C were used in the in vivo CAM assay to evaluate the angiogenic potential and inflammatory response of the materials. This assay evidenced a significant increase in the number of newly formed vessels at the sites of implantation of HAp/MgO spherical granules, when compared to pure HAp granules. Moreover, the inclusion of MgO also resulted in reduced inflammatory response. These favorable results in angiogenesis and inflammation are believed to arise from the release of Mg^2+^ from the HAp/MgO spherical granules, since the role of magnesium ions in improving angiogenesis and reducing inflammation has been reported in other works^63–66^. Particularly, magnesium deficiency inhibits microvascular endothelial cell growth and migration while it increases the synthesis of inflammatory markers. On the contrary, high levels of magnesium stimulate proliferation and sensitize microvascular cells to migratory signals, consequently inducing angiogenesis^64^. An in vitro study also reported that magnesium reduced pro-inflammatory M1 macrophages response when incorporated in a calcium phosphate cement, and the immune microenvironment created by this material also assisted the angiogenesis by HUVEC cells^66^. The results obtained in the CAM model also showed the biocompatibility of the HAp/MgO spherical granules since these materials improved angiogenesis without causing other adverse reactions.

## Conclusions

This work reported a different outcome of incorporating MgO in bone substitutes, particularly in HAp spherical granules. This metal oxide not only provided antibacterial activity but also improved angiogenic response with reduced inflammation, which is a valuable feature for bone regeneration. Moreover, the sintering temperature was also a critical factor for the physico-chemical, mechanical and biological properties of HAp/MgO spherical granules. The best performance was obtained with HAp/MgO spherical granules sintered at 1100 °C that ensured both antibacterial activity towards the three pathogens tested and cytocompatibility with osteoblasts. Hence, these HAp/MgO spherical granules can be a promising antibacterial bone substitute to prevent implant-associated infections.

## Materials and methods

### Synthesis of HAp/MgO spherical granules

The HAp/MgO spherical granules were produced as described elsewhere^22^. Briefly, HAp (nanoXIM·HAp202, FLUIDINOVA, S.A.) and 3 wt% MgO (Alfa Aesar) nanostructured powders were added to a 1.5 wt% sodium alginate (Fisher Scientific) solution in deionized water. The slurry obtained was extruded to a 0.1 M CaCl_2_ using a droplet generation device, instantly forming spheres and left there for 30 min for complete gelation. Pure HAp granules were also produced for comparative studies, using a slurry without MgO (pure HAp). After gelation, spheres were washed with deionized water and the materials were dried overnight at 105 °C. Then, the spheres were sintered for 1 h using three different maximum temperatures: 900, 1100 and 1300 °C. Spherical granules within the range of 0.5–1.0 mm were obtained and their sterilization was performed by autoclaving at 120 °C for 20 min.

### Granules characterization

#### Scanning electron microscopy

Scanning electron microscopy (SEM) was used to observe the surface morphology of the granules. The materials were fixed on aluminium sample holders with carbon tape and sputter coated (SPI Module) with Au/Pd film to provide conductivity. The granules were visualized with a FEI Quanta 400 FEG/ESEM microscope at an acceleration voltage of 15 kV.

#### X-ray diffraction

Phase composition was evaluated by x-ray diffraction (XRD) and the XRD patterns were refined using the Rietveld method. For that purpose, a PANanalytical Empyrean diffractometer was used, with Cu Kα radiation in a 2θ range from 10° to 70°, a step scan time of 48.6410 s and a step size of 0.0260°2θ.

#### Fourier transformed infrared spectroscopy

Chemical characterization of the spherical granules was also performed with Fourier transformed infrared spectroscopy (FTIR). The samples were reduced to powder and analysed as KBr pellets. A Perkin Elmer Frontier FTIR spectrometer was used with a 4 cm^−1^ resolution, a frequency region from 400 to 4000 cm^−1^ and 36 scans accumulated per sample.

#### Mechanical properties (compression strength and friability)

The mechanical properties of the spherical granules were assessed in terms of compression strength. For that purpose, the wells of a 96-well plate were filled with spherical granules, in order to simulate bone defect filling situation. The compression strength was determined using a texture analyser (TA, XT2i, Stable Micro Systems), with a load cell of 30 kg applied vertically and a 4 mm diameter probe. The probe was placed on top of each well and covered a distance of 5 mm at a displacement rate of 0.1 mm/s. The force–time curves were used to determine the compression strength and the analysis was performed in a minimum of 5 individual wells for each sample. The results were expressed as mean ± SD values of 5 individual wells (n = 5).

Additionally, the friability was also studied as described elsewhere^27^ according to a method described in the European Pharmacopeia (5th edition) with minor adaptations. Briefly, 1 g of spherical granules were placed inside a SOTAX/F1 drum that performed 3 cycles of 4 min each operating at 25 RPM. Then, the desegregated powder was discarded with a sieve and the remaining granules were weighted again. Friability was expressed as a percentage of total weight loss.

### Chemical degradation and biodegradation/apatite formation

The chemical degradation test was performed according to ISO 10993-14:2001—“Biological evaluation of medical devices—Part 14: Identification and quantification of degradation products from ceramics” using Tris–HCl buffered solution (pH = 7.4)^67^. The biodegradation and apatite formation was also evaluated using simulated body fluid (SBF) solution. For both tests, spherical granules were immersed in each solution at 37 °C and 120 RPM. After each immersion time (1, 3, 7, 14, 21 and 28 days), the solution was removed and the spherical granules were placed in a vacuum oven until they were complete dried. Weight loss for each material was calculated according to the equation:

$$ {\text{Weight}}\;{\text{loss }}\;\left( \% \right) = \left[ {\left( {{\text{W}}_{0} {-}{\text{ W}}_{{\text{t}}} } \right)/{\text{W}}_{0} } \right] \times {1}00 $$

where W_0_ and W_t_ correspond to initial weight and weight after a certain immersion time, respectively. The solution removed at each immersion time was filtered with a 0.22 µm syringe filter and saved for pH measurements and Mg^2+^ quantification using Induced Coupled Plasma-Atomic Emission Spectroscopy (ICP-AES). The dried spherical granules were analysed with SEM to visualize apatite formation on their surfaces after immersion in SBF. The results were expressed as mean ± SD values of 3 measurements (n = 3).

### Antibacterial activity

#### Planktonic growth

*S. aureus* ATCC 33,591, *S. epidermidis* RP62A and *P. aeruginosa* PAO1 were cultured in tryptic soy broth (Merck) to access the spherical granules potential to reduce bacterial planktonic growth. The materials were placed in 96-well plates and the different bacteria were added at a concentration of 1 × 10^6^ colony-forming units (CFU)/mL. The plate was placed at 37 °C and 150 RPM during 24 h. After incubation, the remaining bacterial suspension was removed, serial diluted in 0.9% NaCl and plated on tryptic soy agar (Liofilchem) during 18 h at 37 °C for colony counting. The results were expressed as the mean ± SD values of five measurements (n = 5).

#### Initial bacterial adhesion

The number of bacteria adhered onto the spherical granules was quantified to evaluate the effect of surface topography and chemical composition on bacterial adherence. For that purpose, granules were placed in 96-well plates and incubated with *S. aureus* ATCC 33,591, *S. epidermidis* RP62A and *P. aeruginosa* PAO1 at 37 °C and 150 RPM, using a concentration of 1 × 10^8^ CFU/mL. After 1 h of incubation, granules were washed with 0.9% NaCl to remove non adherent bacteria. Spherical granules were then transferred to a new 96-well plate and 200 µL of 0.9% NaCl were added to each well. The plate was placed in an ultrasonic bath (Bandelin Sonoprex Digitec) with a frequency of 35 kHz for 15 min to remove the adherent bacteria from the material. The resulting solutions were serial diluted and placed onto tryptic soy agar (Liofilchem) plates, which were incubated at 37 °C for 18 h. Colony counting was performed in the next day and the number of adherent bacteria was expressed in CFU/mL. The results were expressed as the mean ± SD values of five measurements (n = 5).

### In vitro cytocompatibility assessment

The cytocompatibility of the spherical granules was assessed using an osteoblastic cell line derived from mouse calvaria (MC3T3-E1, Gibco). The cells were maintained in alpha minimum essential medium (Gibco) supplemented with 10% foetal bovine serum (Gibco) and 1% penicillin–streptomycin (Gibco). Cultures were incubated in a humidified environment at 37 °C and 5% CO_2_. After cell confluence, cells were seeded on top of the spherical granules at 6 × 10^4^ cells/cm^2^, to evaluate their response to the materials. Cells grown directly on tissue culture plates (TCPS) in the absence of the spherical granules were used as controls.

#### Metabolic activity

The cells metabolic activity was evaluated using the resazurin assay. With that purpose, 10% v/v resazurin was added to the cells and incubated during 4 h. After incubation, 100 µL were transferred to a black 96-well plate and fluorescence was quantified with a microplate reader (SynergyMix, BioTek) at 530 nm excitation and 590 nm emission wavelength. The results were expressed in relative fluorescence units (RFU) as the mean ± SD values of five measurements (n = 5).

#### Cell adhesion and morphology

To observe cells morphology and adherence onto the spherical granules, cells were fixed during 15 min with 4% paraformaldehyde. Then, cells were dehydrated in graded ethanol solutions and hexamethyldisilazane, with concentrations from 50 to 100%. Samples were dried overnight at room temperature and observed with SEM as previously described.

### In vivo chorioallantoic membrane (CAM) assay

The in vivo CAM assay was performed according to the protocol optimized in our institute and previously described^68^. Briefly, chick (*Gallus gallus*) eggs acquired from commercial sources were incubated horizontally at 37.8 °C in a humidified atmosphere and referred to embryonic day (E). On E3 a square window was opened in the shell after removal of 2–2.5 mL of albumen to allow detachment of the developing CAM. The window was sealed with a transparent adhesive tape and the eggs returned to the incubator. At E10, the spherical granules were placed pairwise by sintering temperature on top the growing CAM into a 3 mm silicon ring, under sterile conditions. The eggs were re-sealed and returned to the incubator for additional 3 days. CAMs were then excised from the embryos and photographed *ex ovo* under a stereoscope (Olympus, SZX16 coupled with a DP71 camera) at 20 × magnification. The number of new vessels (< 20 µm in diameter) growing radially towards the inoculation site was counted in a blind fashion manner. Inflammation was assessed semi-quantitatively by a scoring blind system of macroscopic images of excised CAMs [Score 0 = no inflammatory reaction. Score 1 = with inflammatory reaction. Score 2 = with inflammatory reaction, bigger than the counterpart (in the same egg)]. Excised CAMs were fixed in 10% v/v neutral-buffered formalin in PBS, paraffin-embedded for slide sections and stained with haematoxylin–eosin for histological validation of the inflammatory score. Three independent experiments were conducted with a total of 16 pairs (n = 16) for spherical granules sintered at 1100 °C and 14 pairs (n = 14) for spherical granules sintered at 1300 °C. All the experiments using chick embryos were carried out in accordance with the Directive 2010/63/EU of the European parliament and of the council (22 September 2010) on the protection of animals used for scientific purposes. Under the Portuguese National Regulation (Decreto-Lei n.º 113/2013), no approval from Ethics Committee of the Portuguese Official Authority on Animal Welfare and Experimentation (DGAV) was required for the CAM assay presented in this paper since the avian embryos were used before the last third of their normal development (before embryonic day 15).

### Statistical analysis

For all the in vitro tests, the statistical analysis was performed using the two-way analysis of variance (ANOVA) followed by a Tukey’s and Šidák’s multiple comparison tests. Considering the in vivo CAM assay angiogenic response, statistically significant differences between the two conditions (HAp or HAp/MgO), at each temperature (1100 or 1300 °C) tested in the same egg, were analysed using a Wilcoxon matched-pairs signed rank test. To compare the angiogenic response between the two temperatures, a Mann Whitney test was used. For analysis of the inflammatory scores, Chi-square test was applied. All statistical analysis was performed using GraphPad Software version 8 and data were expressed as mean ± standard deviation (SD). Statistical significant differences were indicated with *p < 0.05, **p < 0.01 and ****p < 0.0001.

## Data Availability

The datasets generated during and/or analysed during the current study are available from the corresponding author on reasonable request.

## References

[1] Saladin KS (2004). Anatomy & Physiology: The Unity of Form and Function.

[2] García-Gareta E, Coathup MJ, Blunn GW (2015). Osteoinduction of bone grafting materials for bone repair and regeneration. Bone.

[3] Giannoudis PV, Dinopoulos H, Tsiridis E (2005). Bone substitutes: An update. Injury.

[4] Haugen HJ, Lyngstadaas SP, Rossi F, Perale G (2019). Bone grafts: which is the ideal biomaterial?. J. Clin. Periodontol..

[5] Amini AR, Laurencin CT, Nukavarapu SP (2012). Bone tissue engineering: recent advances and challenges. Crit. Rev. Biomed. Eng..

[6] Bohner M (2010). Resorbable biomaterials as bone graft substitutes. Mater. Today.

[7] Suchanek W, Yoshimura M (1998). Processing and properties of hydroxyapatite-based biomaterials for use as hard tissue replacement implants. J. Mater. Res..

[8] Barradas AM, Yuan H, van Blitterswijk CA, Habibovic P (2011). Osteoinductive biomaterials: current knowledge of properties, experimental models and biological mechanisms. Eur. Cell Mater..

[9] Low KL (2010). Calcium phosphate-based composites as injectable bone substitute materials. J. Biomed. Mater. Res..

[10] Arciola CR, Campoccia D, Ehrlich GD, Montanaro L, Donelli G (2015). Biofilm-based Healthcare-Associated Infections.

[11] Montanaro L (2011). Scenery of Staphylococcus implant infections in orthopedics. Future Microbiol..

[12] Magill SS (2014). Multistate point-prevalence survey of health care-associated infections. N. Engl. J. Med..

[13] Campoccia D, Montanaro L, Arciola CR (2006). The significance of infection related to orthopedic devices and issues of antibiotic resistance. Biomaterials.

[14] Arciola CR, Campoccia D, Montanaro L (2018). Implant infections: adhesion, biofilm formation and immune evasion. Nat. Rev. Microbiol..

[15] Zimmerli W, Moser C (2012). Pathogenesis and treatment concepts of orthopaedic biofilm infections. FEMS Immunol. Med. Microbiol..

[16] Campoccia D, Montanaro L, Speziale P, Arciola CR (2010). Antibiotic-loaded biomaterials and the risks for the spread of antibiotic resistance following their prophylactic and therapeutic clinical use. Biomaterials.

[17] Aslam B (2018). Antibiotic resistance: a rundown of a global crisis. Infect Drug Resist.

[18] Dizaj SM, Lotfipour F, Barzegar-Jalali M, Zarrintan MH, Adibkia K (2014). Antimicrobial activity of the metals and metal oxide nanoparticles. Mater. Sci. Eng., C.

[19] Khan ST, Musarrat J, Al-Khedhairy AA (2016). Countering drug resistance, infectious diseases, and sepsis using metal and metal oxides nanoparticles: current status. Colloids Surf. B.

[20] He Y (2016). Study on the mechanism of antibacterial action of magnesium oxide nanoparticles against foodborne pathogens. J. Nanobiotechnol..

[21] Leung YH (2014). Mechanisms of antibacterial activity of MgO: non-ROS mediated toxicity of MgO nanoparticles towards *Escherichia coli*. Small.

[22] Coelho CC, Araújo R, Quadros PA, Sousa SR, Monteiro FJ (2019). Antibacterial bone substitute of hydroxyapatite and magnesium oxide to prevent dental and orthopaedic infections. Mater. Sci. Eng. C.

[23] Wu L, Feyerabend F, Schilling AF, Willumeit-Römer R, Luthringer BJC (2015). Effects of extracellular magnesium extract on the proliferation and differentiation of human osteoblasts and osteoclasts in coculture. Acta Biomater..

[24] Wetteland CL, Nguyen N-YT, Liu H (2016). Concentration-dependent behaviors of bone marrow derived mesenchymal stem cells and infectious bacteria toward magnesium oxide nanoparticles. Acta Biomater..

[25] Krishnamoorthy K, Moon JY, Hyun HB, Cho SK, Kim S-J (2012). Mechanistic investigation on the toxicity of MgO nanoparticles toward cancer cells. J. Mater. Chem..

[26] Campoccia D, Montanaro L, Arciola CR (2013). A review of the biomaterials technologies for infection-resistant surfaces. Biomaterials.

[27] Oliveira SM (2008). Injectability of a bone filler system based on hydroxyapatite microspheres and a vehicle with in situ gel-forming ability. J. Biomed. Mater. Res. B.

[28] Bohner M (2013). Synthesis of spherical calcium phosphate particles for dental and orthopedic applications. Biomatter.

[29] Wu P-Y (2016). Comparative study on arsenate removal mechanism of MgO and MgO/TiO2 composites: FTIR and XPS analysis. New J. Chem..

[30] Pugazhendhi A, Prabhu R, Muruganantham K, Shanmuganathan R, Natarajan S (2019). Anticancer, antimicrobial and photocatalytic activities of green synthesized magnesium oxide nanoparticles (MgONPs) using aqueous extract of Sargassum wightii. J. Photochem. Photobiol. B.

[31] Bernhardt A, Dittrich R, Lode A, Despang F, Gelinsky M (2013). Nanocrystalline spherical hydroxyapatite granules for bone repair: in vitro evaluation with osteoblast-like cells and osteoclasts. J. Mater. Sci. Mater. Med..

[32] Ribeiro CC, Barrias CC, Barbosa MA (2006). Preparation and characterisation of calcium-phosphate porous microspheres with a uniform size for biomedical applications. J. Mater. Sci. - Mater. Med..

[33] Ginebra MP, Espanol M, Montufar EB, Perez RA, Mestres G (2010). New processing approaches in calcium phosphate cements and their applications in regenerative medicine. Acta Biomater..

[34] Misiek DJ, Kent JN, Carr RF (1984). Soft tissue responses to hydroxylapatite particles of different shapes. J. Oral Maxillofac. Surg..

[35] Gautam CR, Kumar S, Biradar S, Jose S, Mishra VK (2016). Synthesis and enhanced mechanical properties of MgO substituted hydroxyapatite: a bone substitute material. RSC Adv..

[36] Yuan Z (2019). Injectable PLGA microspheres with tunable magnesium ion release for promoting bone regeneration. Acta Biomater..

[37] Hung C-C, Chaya A, Liu K, Verdelis K, Sfeir C (2019). The role of magnesium ions in bone regeneration involves the canonical Wnt signaling pathway. Acta Biomater..

[38] Mejias JA, Berry AJ, Refson K, Fraser DG (1999). The kinetics and mechanism of MgO dissolution. Chem. Phys. Lett..

[39] Mueller W-D, Lorenzo F, de Mele M, Nascimento ML, Zeddies M (2009). Degradation of magnesium and its alloys: dependence on the composition of the synthetic biological media. J. Biomed. Mater. Res. A.

[40] Bohner M, Lemaitre J (2009). Can bioactivity be tested in vitro with SBF solution?. Biomaterials.

[41] Wiesmann H-P (1997). Magnesium in newly formed dentin mineral of rat incisor. J. Bone Mier. Res..

[42] Bigi A (1993). Magnesium influence on hydroxyapatite crystallization. J. Inorg. Biochem..

[43] Barrere F, van Blitterswijk CA, de Groot K, Layrolle P (2002). Nucleation of biomimetic Ca–P coatings on Ti6Al4V from a SBF×5 solution: influence of magnesium. Biomaterials.

[44] Dietrich E, Oudadesse H, Lucas-Girot A, Mami M (2009). In vitro bioactivity of melt-derived glass 46S6 doped with magnesium. J. Biomed. Mater. Res., Part A.

[45] Chou Y-F, Chiou W-A, Xu Y, Dunn JCY, Wu BM (2004). The effect of pH on the structural evolution of accelerated biomimetic apatite. Biomaterials.

[46] Sawai J (2003). Quantitative evaluation of antibacterial activities of metallic oxide powders (ZnO, MgO and CaO) by conductimetric assay. J. Microbiol. Methods.

[47] Yamamoto O, Ohira T, Alvarez K, Fukuda M (2010). Antibacterial characteristics of CaCO3–MgO composites. Mater. Sci. Eng. B.

[48] Vidic J (2013). Selective antibacterial effects of mixed ZnMgO nanoparticles. J. Nanopart. Res..

[49] Aničić N, Vukomanović M, Suvorov D (2016). The nano-texturing of MgO microrods for antibacterial applications. RSC Adv..

[50] Li Y, Zhang W, Niu J, Chen Y (2012). Mechanism of Photogenerated Reactive Oxygen Species and Correlation with the Antibacterial Properties of Engineered Metal-Oxide Nanoparticles. ACS Nano.

[51] Luque-Agudo V (2020). The role of magnesium in biomaterials related infections. Colloids Surf., B.

[52] Sawai J (2000). Antibacterial characteristics of magnesium oxide powder. World J. Microbiol. Biotechnol..

[53] Hetrick EM, Schoenfisch MH (2006). Reducing implant-related infections: active release strategies. Chem. Soc. Rev..

[54] Ribeiro M, Monteiro FJ, Ferraz MP (2012). Infection of orthopedic implants with emphasis on bacterial adhesion process and techniques used in studying bacterial-material interactions. Biomatter.

[55] Bryers JD (2008). Medical biofilms. Biotechnol. Bioeng..

[56] Grenho L, Manso MC, Monteiro FJ, Ferraz MP (2012). Adhesion of Staphylococcus aureus, Staphylococcus epidermidis, and Pseudomonas aeruginosa onto nanohydroxyapatite as a bone regeneration material. J. Biomed. Mater. Res., Part A.

[57] Ribeiro M, Monteiro FJ, Ferraz MP (2012). Staphylococcus aureus and Staphylococcus epidermidis adhesion to nanohydroxyapatite in the presence of model proteins. Biomed. Mater..

[58] Lerebour G, Cupferman S, Bellon-Fontaine MN (2004). Adhesion of Staphylococcus aureus and Staphylococcus epidermidis to the Episkin® reconstructed epidermis model and to an inert 304 stainless steel substrate. J. Appl. Microbiol..

[59] Predoi D, Iconaru SL, Predoi MV, Stan GE, Buton N (2019). Synthesis, characterization, and antimicrobial activity of magnesium-doped hydroxyapatite suspensions. Nanomaterials.

[60] Ghobadian M, Nabiuni M, Parivar K, Fathi M, Pazooki J (2015). Toxic effects of magnesium oxide nanoparticles on early developmental and larval stages of zebrafish (Danio rerio). Ecotoxicol. Environ. Saf..

[61] Pourdanesh F, Jebali A, Hekmatimoghaddam S, Allaveisie A (2014). In vitro and in vivo evaluation of a new nanocomposite, containing high density polyethylene, tricalcium phosphate, hydroxyapatite, and magnesium oxide nanoparticles. Mater. Sci. Eng. C.

[62] Roh H-S (2017). Addition of MgO nanoparticles and plasma surface treatment of three-dimensional printed polycaprolactone/hydroxyapatite scaffolds for improving bone regeneration. Mater. Sci. Eng., C.

[63] Maier JAM, Bernardini D, Rayssiguier Y, Mazur A (2004). High concentrations of magnesium modulate vascular endothelial cell behaviour in vitro. Biochim. Biophys. Acta.

[64] Bernardini D, Nasulewic A, Mazur A, Maier JAM (2005). Magnesium and microvascular endothelial cells: a role in inflammation and angiogenesis. Front. Biosci..

[65] Mazur A (2007). Magnesium and the inflammatory response: Potential physiopathological implications. Arch. Biochem. Biophys..

[66] Wang M (2016). Improved osteogenesis and angiogenesis of magnesium-doped calcium phosphate cement via macrophage immunomodulation. Biomater. Sci..

[67] ISO 10993-14:2001 *Biological Evaluation of Medical Devices—Part 14: Identification and Quantification of Degradation Products from Ceramics*. (International Organization of Standardization, Geneva 2001).

[68] Loureiro J (2019). Conjugation of the T1 sequence from CCN1 to fibrin hydrogels for therapeutic vascularization. Mater. Sci. Eng., C.

